# Concomitant Prevalence of Low Serum Diamine Oxidase Activity and Carbohydrate Malabsorption

**DOI:** 10.1155/2016/4893501

**Published:** 2016-11-30

**Authors:** Dietmar Enko, Andreas Meinitzer, Harald Mangge, Gernot Kriegshäuser, Gabriele Halwachs-Baumann, Eva Z. Reininghaus, Susanne A. Bengesser, Wolfgang J. Schnedl

**Affiliations:** ^1^Institute of Clinical Chemistry and Laboratory Medicine, General Hospital Steyr, Steyr, Austria; ^2^Department of Gastroenterology, General Hospital Steyr, Steyr, Austria; ^3^Clinical Institute of Medical and Laboratory Diagnostics, Medical University of Graz, Graz, Austria; ^4^Department of Psychiatry and Psychotherapeutic Medicine, Medical University of Graz, Graz, Austria; ^5^Practice for General Internal Medicine, Bruck an der Mur, Austria; ^6^Medical University of Graz, Graz, Austria

## Abstract

The aim of this retrospective study was to analyze the concomitant prevalence rates for lactose malabsorption (LM), fructose malabsorption (FM), and histamine intolerance (HI) in patients with so far unexplained gastrointestinal (GI) symptoms. A total of 439 outpatients, who presented unclear abdominal discomfort, underwent lactose (50 g) and fructose (25 g) hydrogen (H_2_) breath tests. Additionally, serum diamine oxidase (DAO) measurements were performed. Individuals with low serum DAO activity (<10 U/mL), GI symptoms, and response to histamine-free diet were diagnosed with HI. Of all 439 patients, 341 (77.7%) were found with 7 various GI conditions. In total, 94 (21.4%), 31 (7.1%), and 100 (22.8%) individuals presented LM, FM, or HI only, whereas 116 (26.4%) patients showed an overlap of GI entities investigated here. Interestingly, 89 out of 241 (36.9%) individuals with carbohydrate malabsorption were also diagnosed with HI (LM + HI: 52 [11.8%], FM + HI: 23 [5.2%], and LM + FM + HI 14 [3.2%] individuals). In conclusion different combinations of LM, FM, and HI are present in individuals with unclear abdominal discomfort/pain. In clinical practice we suggest testing for LM, FM, and additional HI in the diagnostic work-up of these patients. Depending on these various diagnoses possible, patients should get an individualized dietary advice.

## 1. Introduction

Nonspecific abdominal discomfort is a common and widespread condition worldwide. Among the multitude of differential diagnoses, carbohydrate malabsorption is a frequent cause of unexplained gastrointestinal (GI) symptoms [1–3].

Lactose malabsorption (LM) results from a reduced expression or impaired activity of the enzyme lactase in the epithelium of the small intestine. Hence, the nonabsorbable disaccharide lactose cannot be cleaved into the absorbable monosaccharides glucose and galactose [3, 4]. By comparison, fructose malabsorption (FM) is caused by limited absorption capacity of the GLUT-5 protein, the major fructose transporter in the small intestine [3, 5, 6]. The undigested and unabsorbed carbohydrates reach the large intestine, where the colonic bacteria ferment the sugar molecules, which may cause GI symptoms such as abdominal pain, bloating, and/or diarrhea [3, 7].

These complaints are also the leading GI symptoms of histamine intolerance (HI) [8, 9]. Therefore, this GI condition should also be considered as a possible underlying reason of unclear abdominal discomfort [8]. HI results from a disequilibrium of the biogenic amine histamine, which occurs to various degrees in many foods, and the reduced capacity of histamine degradation. An impaired activity (<10 U/mL) of serum diamine oxidase (DAO), which is the main enzyme for the metabolism of ingested histamine, causes an insufficient extracellular histamine breakdown in the GI tract [8, 10].

Using MEDLINE® database (US National Library of Medicine, Bethesda, MD, USA), we could not find studies investigating serum DAO measurements in patients with carbohydrate malabsorption. Therefore, this retrospective study was conducted to investigate concomitant prevalence rates of LM, FM, and HI in patients presenting unexplained abdominal discomfort.

## 2. Materials and Methods

### 2.1. Patients

A total of 439 case histories of outpatients, who visited the medical practice of internal medicine due to nonspecific abdominal complaints, were included in this retrospective analysis. The ethnic origin of all included individuals was Caucasian. All of them were investigated for LM, FM, and HI. Blood draw from a peripheral vein for DAO activity measurements was performed in the morning after an overnight fasting state (>12 h). None of the included patients was on histamine-free diet, had histamine releasing drugs, or had alcohol at the time of blood sampling and anamnesis. At the presentation a thorough evaluation of abdominal symptoms was made including a dietary history and the association between meal consumption and the occurrence of symptoms (Table 1).

Individuals presenting extraintestinal symptoms (e.g., migraine, dizziness, malaise, or headache) only, patients with gastritis caused by the intake of nonsteroidal anti-inflammatory drugs, patients with acute or chronic inflammatory bowel or infectious disease and individuals with elevated anti-tissue-transglutaminase (TTG) IgA antibodies were not included in this evaluation. In addition to positive TTG IgA testing, a biopsy-based histological investigation of the small intestinal mucosa was part of the diagnostic evaluation.* Helicobacter pylori* infection was tested in all patients and eradication therapy was initiated if applicable. The data on* H. pylori *were not included in this manuscript because a causative role in GI malabsorption comparable to carbohydrate malabsorption is not confirmed.

This study was approved by the Ethical Committee of the Johannes Kepler University Linz (Linz, Austria) and is in accordance with the latest version of the Declaration of Helsinki.

### 2.2. LM and FM

Gas chromatography (Gastrolyzer, Bedfont Scientific Inc., Kent, United Kingdom) was employed to detect patients with LM and FM. Baseline breath hydrogen (H_2_) levels were measured after an overnight fasting state of > 12 hours. Lactose was given in a dose of 50 g dissolved in 200 mL of water. In the course of a following visit fructose was given in a dose of 25 g dissolved in 200 mL of water. In both tests the end-expiratory breath H_2_ concentration was measured at 0 (baseline before sugar ingestion), 30, 60, 90, and 120 minutes. The results were expressed in parts per million (ppm). According to the literature [3, 11, 12], patients with a H_2_ peak > 20 ppm above baseline H_2_ concentrations were classified as lactose and/or fructose malabsorbers independently of their self-reported clinical symptoms during or after the breath test. Patients were instructed to avoid physical effort, smoking, or eating during the lactose and fructose breath test. Patients with a colonoscopy, an antibiotic- or a laxatives-based therapy at least two weeks before test procedures, were excluded from the breath testing.

### 2.3. HI

The serum diamine oxidase (DAO) activity was measured using a quantitative radio extraction assay (DAO-REA®; Sciotec Diagnostic Technologies GmbH, Tulln, Austria).

The intra- and interassay reproducibility of the DAO radio extraction assay was < 10 and < 15% (information of the manufacturer). According to the literature [8, 13], in individuals with serum DAO activity < 3 U/mL, HI intolerance was expected, while in patients with serum DAO levels between 3 and 10 U/mL, HI was considered possible. Patients with DAO levels < 10 U/mL were identified with HI only if they showed two or more GI symptoms of HI (e.g., nausea, vomiting, meteorism, and/or abdominal pain) and a positive response to a low histamine diet [8, 13].

### 2.4. Statistics

Descriptive statistics were performed to analyze prevalence rates for LM, FM, and HI. The exact Chi-Square test for independence was used to compare categorical variables. A *p*-value < 0.05 was considered statistically significant. SPSS Statistics for Windows version 22.0 (IBM SPSS Inc., Chicago, Illinois, USA) was used for statistical analysis.

## 3. Results

### 3.1. Patient Population Characteristics

Of all 439 patients included, 138 (31.4%) were male and 301 (68.6%) were female with a median age of 43.8 years. The main demographic and clinical characteristics of the study population including the assessment of GI symptoms during the first consultation are provided in Table 1. None of the included patients showed signs of active acute or chronic inflammatory bowel disease.

### 3.2. DAO Serum Levels

All in all, 51 individuals (11.6%) were identified with a serum DAO level < 3 U/mL, 138 patients (31.4%) had a serum DAO level between 3 and 10 U/mL, and 250 (57.0%) individuals showed DAO serum levels ≥ 10 U/mL. No correlation was observed between serum DAO levels and the occurrence of LM (*p* = 0.395) or FM (*p* = 0.615).

### 3.3. Differential Diagnoses of GI Conditions

As shown in Table 2, 341 (77.7%) patients were found with 7 various GI conditions, while 98 (22.3%) individuals remained negative for any GI entity investigated here. All in all, 94 (21.4%), 31 (7.1%), and 100 (22.8%) individuals presented LM, FM, or HI only, whereas 116 (26.4%) showed a diagnostic overlap of LM, FM and HI, respectively. Interestingly, 89 out of 241 (36.9%) individuals with carbohydrate malabsorption were identified with HI.

## 4. Discussion

In this study, 341 (77.7%) individuals with unclear abdominal complaints presented 7 various GI conditions. To the best of our knowledge, this is the most extensive investigation on concomitant prevalence rates for LM, FM, and including HI in such a large collective of patients.

With respect to carbohydrate malabsorption, H_2_ breath testing identified 241 (54.9%) patients with LM and/or FM, of which 89 (36.9%) individuals were also diagnosed with HI. These findings demonstrate that multiple combinations of carbohydrate malabsorption with HI may be associated with GI discomfort. This seems a great challenge for clinicians in view of individualized treatment options. Individuals with LM, FM and/or HI should get detailed dietary advice by registered dietitians, preferably at time of diagnosis.

In the present study and according to the literature [8, 13], individuals with serum DAO activity < 3 U/mL were expected with HI, while in patients with serum DAO levels between 3 and 10 U/mL, HI was considered possible. We observed no correlation between serum DAO levels and the occurrence of LM (*p* = 0.395) or FM (*p* = 0.615). Nevertheless, present data show, that the diagnostic overlap between LM and HI was higher compared to FM and HI. In a recent study, we found serum DAO activity associated with LM phenotypic variation [14]. We observed, that patients with LM and a serum DAO activity level < 10 U/L presented higher end-exspiratory H_2_-levels in the lactose breath test compared to LM patients with DAO activity levels ≥ 10 U/mL [14]. DAO, the main enzyme in metabolizing ingested histamine, is synthesized by mature apical enterocytes, which are located in the upper intestinal villi [15]. It is continuously released from the intestinal mucosa and also transported to the blood circulation [16]. Mucosal damage in the small intestine caused by GI conditions (e.g., gastroenteritis, short bowel syndrome, GI surgery, drugs, celiac and tropical sprue) may reduce DAO and lactase activity, respectively [14, 17]. This could be one possible reason for the diagnostic overlap between LM and HI observed here.

The diagnosis of HI has its limitations. In general, the diagnostic approach of HI is difficult because the symptoms are highly variable and may affect almost all organs [9]. Moreover, standardized* in vitro* diagnostic tests for HI testing are still lacking [18]. Therapy of HI should be based on a consequent avoidance of histamine-rich food (e.g., spinach, tomatoes, long-ripened or fermented products, alcohol) or histamine liberators (e.g., citrus fruits, chocolate). A diet diary is suggested to document the improvement of symptoms during a histamine-free diet and to record the relapses in HI after dietary mistakes [8].

Not only the diagnostic approach of HI is difficult, but also limitations of the conventional lactose and fructose H_2_ breath test must be mentioned. The functional H_2_ breath test is considered a reliable, non-invasive diagnostic approach for patients with LM and FM [19], but the method is not standardized yet and pre-analytical poor patient preparation during the alveolar air collection may lead to false-negative test results [12]. Moreover, the acidic microclimate in the colon of patients with carbohydrate malabsorption can also cause decreased bacterial H_2_ production [20]. Another influencing cofactor of the H_2_ breath test is the orocecal transit time, which may also lead to false negative test results, because breath testing may be finished before a measurable H_2_ increase is established [11, 12]. Furthermore an individual and subjective perception of GI symptoms, which patients associate with carbohydrate malabsorption, must be considered [12, 21]. Visceral hypersensitivity is considered to play an essential role in functional symptoms, but the causal symptom triggers are not completely understood yet [3, 19].

Two limitations of this study may be described. Firstly, activities of the intestinal histamine-degrading enzyme histamine N-methyl-transferase were not measured because no standardized kit is commercially available yet. Secondly, serum DAO levels were determined at a single measuring point, only. Therefore, a prospective longitudinal study containing follow-up measurements is required to assess the clinical course of serum DAO levels in patients with unclear abdominal discomfort.

## 5. Conclusions

Patients with unexplained abdominal discomfort/pain present multiple combinations of carbohydrate malabsorption and HI. As a consequence we suggest testing for LM, FM, and additionally HI in the diagnostic work-up of these patients in clinical practice. Depending on differential diagnostic considerations, patients should get an individualized dietary advice.

## Figures and Tables

**Table 1:** Main demographic and clinical characteristics of the study population.

	Study population
Patients	439 (100.0%)
Gender	
Male	138 (31.4%)
Female	301 (68.6%)
Median age, years (25th, 75th percentile)	43.8 (29.9, 55.4)
Presence of gastrointestinal symptoms	
Abdominal pain/discomfort	99 (22.6%)
Bloating/flatulence	299 (68.1%)
Diarrhea	249 (56.7%)
Obstipation	24 (5.5%)
Others (nausea, emesis, burping, and heartburn)	61 (13.9%)

**Table 2:** Prevalence rates of LM, FM, and HI.

GI conditions	Patients	Percent
LM	94	21.4
FM	31	7.1
LM + FM	27	6.2
HI	100	22.8
LM + HI	52	11.8
FM + HI	23	5.2
LM + FM + HI	14	3.2

Total	341	77.7

GI: gastrointestinal; LM: lactose malabsorption; FM: fructose malabsorption; HI: histamine intolerance.
